# Veno-Arterial Extracorporeal Membrane Oxygenation in Elective High-Risk Percutaneous Coronary Interventions

**DOI:** 10.3389/fmed.2022.913403

**Published:** 2022-05-26

**Authors:** Ming Bai, Andong Lu, Chenliang Pan, Sixiong Hu, Wenjing Qu, Jing Zhao, Bo Zhang

**Affiliations:** ^1^The First School of Clinical Medicine of Lanzhou University, Lanzhou, China; ^2^Heart Center, The First Hospital of Lanzhou University, Lanzhou, China; ^3^Gansu Key Laboratory for Cardiovascular Diseases of Gansu Province, Lanzhou, China; ^4^Cardiovascular Clinical Research Center of Gansu Province, Lanzhou, China

**Keywords:** veno-arterial extracorporeal membrane oxygenation, elective high-risk percutaneous coronary interventions, safe and feasible, complications, outcomes

## Abstract

**Background:**

The safety and feasibility of veno-arterial extracorporeal membrane oxygenation (VA-ECMO) as mechanical circulatory support in high-risk percutaneous coronary intervention (HR-PCI) remain unclear.

**Methods:**

This retrospective study included patients with complex and high-risk coronary artery disease who underwent elective PCI with VA-ECMO support pre-operatively during March 2019–December 2020. Rates of VA-ECMO-related complications, complications during PCI, death, myocardial infarction, and stroke during hospitalisation and 1-year post-operatively were analysed.

**Results:**

Overall, 36 patients (average age: 63.6 ± 8.9 years) underwent PCI. The average duration of VA-ECMO support was 12.5 (range, 3.0–26.3) h. Intra-aortic balloon pump counterpulsation was used in 44.4% of patients. The SYNTAX score was 34.6 ± 8.4 pre-operatively and 10.8 ± 8.8 post-operatively (*P* < 0.001). Intraoperative complications included pericardial tamponade (*N* = 2, 5.6%), acute left-sided heart failure (*N* = 1, 2.8%), malignant arrhythmia requiring electrocardioversion (*N* = 2, 5.6%), and no deaths. Blood haemoglobin levels before PCI and 24 h after VA-ECMO withdrawal were 145.4 ± 20.2 g/L and 105.7 ± 21.7 g/L, respectively (*P* < 0.001). Outcomes during hospitalisation included death (*N* = 1, 2.8%), stroke (*N* = 1, 2.8%), lower limb ischaemia (*N* = 2, 5.6%), lower limb deep venous thrombosis (*N* = 1, 2.8%), cannulation site haematoma (*N* = 2, 5.6%), acute renal injury (*N* = 2, 5.6%), bacteraemia (*N* = 2, 5.6%), bleeding requiring blood transfusion (*N* = 5, 13.9%), and no recurrent myocardial infarctions. Within 1 year post-operatively, two patients (5.6%) were hospitalised for heart failure.

**Conclusions:**

Veno-arterial extracorporeal membrane oxygenation mechanical circulation support during HR-PCI is a safe and feasible strategy for achieving revascularisation in complex and high-risk coronary artery lesions. VA-ECMO-related complications require special attention.

## Introduction

Elective revascularisation procedures for coronary artery disease include percutaneous coronary intervention (PCI) and coronary artery bypass grafting (CABG). While guidelines recommend CABG as the procedure of choice in patients with complex and high-risk coronary artery disease, PCI usage as a revascularisation strategy is increasing in patients who are not suitable for CABG (1–4). While revascularisation (PCI or CABG) can improve the prognosis of patients with complex coronary artery lesions (5, 6), the rate of revascularisation in these patients is low (7, 8). Complex and high-risk coronary artery disease lesions can be revascularised using high-risk PCI (HR-PCI). However, HR-PCI is associated with various complications, such as no coronary artery reflow, coronary artery dissection, pericardial tamponade haemodynamic instability, and cardiac arrest (3). Therefore, HR-PCI represents challenge for interventional cardiologists. The current literature suggests that this type of revascularisation can be completed with mechanical circulation support (3, 4, 9). Mechanical circulation support devices used during HR-PCI include intra-aortic balloon pump (IABP) counterpulsation, extracorporeal membrane oxygenation (ECMO), Impella (Abiomed, Danvers, MA, United States), and TandemHeart (LivaNova Medical Technology Co., Ltd., Pittsburgh, PA, United States) devices (4, 10). Since haemodynamic instability or cardiac arrest can occur during HR-PCI, ECMO can provide strong circulatory support and significantly improve patient prognosis (11). However, veno-arterial (VA)-ECMO can increase the risk of complications, such as cardiac afterload, lower limb arterial ischaemia, blood cell destruction, and increased risk of infection (10). Since clinical data using VA-ECMO as mechanical circulation support in HR-PCI are currently lacking, no recommendations are included in the guidelines. This study aimed to analyse the results of the prophylactic use of VA-ECMO during HR-PCI.

## Materials and Methods

### Study Population and Design

This single-centre retrospective observational study included 36 patients who underwent elective HR-PCI with VA-ECMO support between March 2019 and December 2020. Patients ranged in age from 18 to 85 years. Indications of VA-ECMO use in patients were: (1) left ventricular ejection fraction (LVEF) ≤35%; (2) LVEF >35%; simultaneous merging with the following criteria: (a) coronary artery calcification that requires rotary grinding, (b) unprotected left main coronary artery, and (c) coronary arteries with two chronic total occlusions (CTOs) and one severe stenosis. Indications in these patients included unstable angina pectoris, acute myocardial infarction, and old myocardial infarction. Patients with cardiogenic shock were not included. All patients underwent implantation of ECMO before PCI, and the VA-ECMO mode was chosen. The common femoral artery and vein were selected for ECMO approach in all the patients. An arterial cannulae at 15–19 French (F) and a venous cannulae at 19–21F were chosen as they are 1–2 mm smaller than the inner diameter of the blood vessel. Distal perfusion ipsilateral to the femoral artery cannulation was performed with a 6F catheter. All cannulations were performed under ultrasound guidance. Heparin (100 units/kg before ECMO insertion) was used as anticoagulation strategy. The activated clotting time was maintained for 180–200 s during ECMO insertion and for 250–350 s during PCI. All patients were given 300 mg aspirin and 180 mg ticagrelor or 300 mg clopidogrel orally before PCI and routinely after PCI. ECMO blood flow was initially set to 2.0 L/min and subsequently adjusted according to a patient's blood pressure.

Patients who had refused CABG were considered for elective HR-PCI after evaluation by the interventional team. Clinical and PCI data, collected by reviewing electronic medical records, included demographic information, comorbidities, characteristics of the coronary artery lesions, and major adverse cardiovascular and cerebrovascular events (MACCEs), such as all-cause mortality, recurrent myocardial infarction, stroke, and hospitalisation due to heart failure. The incidence of follow-up MACCEs after discharge was obtained via telephone interviews. Acute kidney injury (AKI) is defined as any of the following (12): (1) increase in serum creatine (SCr) by ≥0.3 mg/dl (≥26.5 μmol/L) within 48 h; (2) increase in SCr to ≥1.5 times of baseline value, which is known or presumed to have occurred within the prior week; and (3) urine volume <0.5 ml/kg/h for 6 h. According to the fourth edition of the global myocardial infarction, acute myocardial infarction refers to increase and/or decrease of serum cardiac troponin at least 1 time higher than the upper limit of the normal range with concurrent clinical evidence of acute myocardial ischaemia, including (13): (1) symptoms of acute myocardial ischaemia; (2) new ischaemic electrocardiogram changes; (3) new pathogenesis of Q wave; (4) imaging evidence of new viable myocardial loss or abnormal ventricular wall segmental movement; and (5) coronary artery thrombosis confirmation by coronary angiography or intracavitary imaging examination. Coronary artery disease was defined as any of the followings (14): (1) left main coronary artery stenosis ≥50%, (2) one or more main coronary arteries stenosis ≥70%, and (3) microvascular dysfunction and coronary artery spasm that result in exercise and stress-related chest symptoms. CTO refers to coronary artery obstruction with positive Thrombolysis in Myocardial Infarction (TIMI) blood flow level 0 and occlusion time ≥3 months, if there are ipsilateral bridging or ipsilateral collateral vessels, complete occlusion is still considered despite TIMI blood flow level >0 in distal occluded vessels (15). The SYNTAX and European System for Cardiac Operative Risk Evaluation (EuroSCORE) scores were calculated online (http://syntaxscore.org/calculator/start.htm and http://www.euroscore.org/calc.html, respectively). CTO Registry of Japan (J-CTO) scores were calculated for patients with CTO lesions (16). Bleeding events were defined according to the Bleeding Academic Research Consortium (BARC) criteria, and bleeding requiring transfusion was defined as BARC type 3a and 3b bleeding (17). The study protocol, which conformed to the ethical guidelines of the 1975 Declaration of Helsinki, was approved by the Ethics Committee of the First Hospital of Lanzhou University (Approval No. LDYYLL-2021-469). The requirement for informed consent was waived due to the retrospective nature of the study.

### Clinical Outcomes

Clinical outcomes included MACCEs during hospitalisation and within 1 year after PCI. The safety endpoints included lower limb ischaemia, deep venous thrombosis, intubation-related haematoma, pseudoaneurysm, arteriovenous fistula, acute renal injury, bacteraemia, and bleeding requiring blood transfusion.

### Statistical Analyses

All data analyses were performed using STATA V.17 (Stata Corporation, College Station, TX, United States). Metrological data by or approximate to a normal distribution were expressed as means ± standard deviations. If not normally distributed, metrological data were expressed as medians and quartile spacing [M (Q1–Q3)]. Count data were expressed as numbers and/or percentages [*N* (%)]. Normally distributed continuous variables were reported as means ± standard deviations. Continuous variables that were not normally distributed were expressed as M (Q1–Q3). Binary or categorical variables were reported as numbers and percentages [*N* (%)]. A paired *t*-test was used to compare the mean values of the related variables before and after ECMO support. A Kaplan–Meier analysis was performed to plot the survival curves. All tests were two-tailed. Statistical significance was set at *P* < 0.05.

## Results

### Baseline Characteristics

In total, 36 patients (average age, 63.6 ± 8.9 years) were included in this study. Among the patients, 34 (94.4%) were men. Most patients had previous comorbidities. Overall, 61.1% of the patients had LVEF ≥45, 25.0% had LVEF ≤35, 13.9% had a LVEF of 35–45%. Pre-operatively, the haemodynamic parameters of all patients were stable, including those with unstable angina pectoris (58.3%), acute myocardial infarction (19.4%), and old myocardial infarction (22.2%). All patients declined the CABG procedure. The duration of hospital stay was 13.5 (9.0–16.0) days, and the duration of stay in the coronary care unit was 4.5 (3.0–7.5) days. Baseline characteristics are summarised in Table 1.

**Table 1:** Baseline characteristics of patients included in the study.

**Parameter**	**ECMO (*N* = 36)**
Age (years)	63.6 ± 8.9
Sex, male	34 (94.4%)
Current smoker	18 (50.0%)
Hypertension	16 (44.4%)
Diabetes mellitus	12 (33.3%)
Hyperlipidaemia	2 (5.6%)
Prior stroke	1 (2.8%)
Prior MI	7 (19.4%)
Prior CABG	2 (5.6%)
Prior PCI	2 (5.6%)
Early renal insufficiency	1 (2.8%)
Late renal insufficiency	2 (5.6%)
**LVEF (%)**
≤ 35	9 (25.0%)
35–45	5 (13.9%)
≥45	22 (61.1%)
Systolic blood pressure (mmHg)	125.6 ± 24.1
Diastolic blood pressure (mmHg)	74.5 ± 11.5
Heart rate (beats/min)	75.6 ± 14.4
UA	21 (58.3%)
AMI	7 (19.4%)
OMI	8 (22.2%)
Refused surgery	36 (100%)
**Length of stay (days)**
Cardiac care unit	4.5 (3.0–7.5)
Hospital	13.5 (9.0–16.0)

*Data are presented as means ± standard deviations, N (%), or medians (Q1–Q3)*.

*AMI, acute myocardial infarction; CABG, coronary artery bypass grafting; ECMO, extracorporeal membrane oxygenation; eGFR, estimated glomerular filtration rate; LVEDV, left ventricular end-diastolic volume; LVEF, left ventricular ejection fraction; MI, myocardial infarction; OMI, old myocardial infarction; PCI, percutaneous coronary intervention; UA, unstable angina*.

Angiographic and procedural data are summarised in Table 2. The average EuroSCORE I was 7.8 ± 2.3. The pre-PCI and post-PCI SYNTAX scores were 34.6 ± 8.4 and 10.8 ± 8.8, respectively (*P* < 0.001). The most common coronary artery lesions were three-vessel lesions (50.0%). CTO lesions, most of which involved one or two vessels, were present in 77.8% of patients. The average J-CTO score was 1.3 ± 0.9. Coronary artery disease was mainly of the left anterior descending artery (94.4%), followed by the left main coronary artery (41.7%). Additionally, 25.0% of the patients had coronary artery calcification. Revascularisation was performed in two branches in 57% of the patients with an average of 3.0 (2.0–4.0) stents implanted. All HR-PCI procedures were completed successfully. The average duration of ECMO support was 12.5 (3.0–26.3) h. Additional IABP counterpulsation was used in 44.4% of patients. Patients received IABP support mainly for the following reasons: acute left heart failure after the use of VA-ECMO, left ventricular blood stasis indicated by cardiac ultrasound, and contrast agent retention in the coronary sinus during PCI. IABP was also used in patients with poor cardiac function after the weaning from VA-ECMO. Intraoperative complications included pericardial tamponade (*N* = 2, 5.6%), acute left-sided heart failure (*N* = 1, 2.8%), and malignant arrhythmia requiring electrical cardioversion (*N* = 2, 5.6%). None of the patients died during PCI. There were nine patients with significantly reduced intraoperative blood pressure, which was maintained by increasing ECMO blood flow and treating with vasopressor drugs. The lowest intraoperative systolic blood pressure and diastolic blood pressure of these patients were 65.0 ± 11.5 and 47.7 ± 10.7 mmHg. The vasoactive-inotropic score was 39.7 ± 21.2. The maximum intraoperative ECMO blood flow was 3.2 ± 0.3 L/min.

**Table 2:** Angiographic and procedural characteristics of patients included in this study.

**Parameter**	**ECMO (*N* = 36)**
EuroSCORE I	7.8 ± 2.3
**Baseline SYNTAX score**
≤ 22	3 (8.3%)
23–32	9 (25.0%)
≥33	24 (66.7%)
SYNTAX score pre-PCI	34.6 ± 8.4
SYNTAX score post-PCI	10.8 ± 8.8
J-CTO score	1.3 ± 0.9
**Number of diseased vessels**
One vessel	0 (0)
Two vessels	8 (22.2%)
Three vessels	18 (50.0%)
Four or more vessels	10 (27.8)
**Number of vessels treated**
One vessel	8 (22.2%)
Two vessels	20 (55.6%)
Three vessels	6 (16.7%)
Four or more vessels	2 (5.6%)
**Number of CTOs**
One vessel	15 (41.7%)
Two vessels	13 (36.1%)
Three vessels	0 (0)
**Lesion location**
Left anterior descending	34 (94.4%)
Left circumflex	28 (77.7%)
Right coronary artery	28 (77.7%)
Ramus	4 (11.1%)
Left main	15 (41.7%)
Isolated	0 (0)
Plus one vessel	0 (0)
Plus two vessels	5 (13.9%)
Plus three or more vessels	10 (27.8%)
Coronary artery calcification	9 (25.0%)
Number of stents placed	3.0 (2.0–4.0)
Duration of device support (h)	12.5 (3.0–26.3)
Combined IABP counterpulsation	16 (44.4%)
Distal perfusion cannula	2 (5.6%)
Device malfunction	0 (0)
Blood pressure reduction during operation	9 (25.0%)
Lowest systolic blood pressure (mmHg)	65.0 ± 11.5
Lowest diastolic blood pressure (mmHg)	47.7 ± 10.7
Vasoactive-inotropic score	39.7 ± 21.2
Maximum ECMO blood flow (L/min)	3.2 ± 0.3
**MACCE during operation**
Pericardial tamponade	2 (5.6%)
Acute Left ventricular failure	1 (2.8%)
Malignant arrhythmia	2 (5.6%)
Death	0 (0)

*Data are presented as means ± standard deviations, N (%), or medians (Q1–Q3)*.

*CTO, chronic total occlusion; ECMO, extracorporeal membrane oxygenation; IABP, intra-aortic balloon pump; MACCE, major adverse cardiac and cerebrovascular event; PCI, percutaneous coronary intervention*.

Changes in blood cell counts, renal function parameters, and cardiac ultrasound indices are summarised in Table 3. The average blood haemoglobin level was 145.5 ± 19.9 g/L before PCI and 105.5 ± 21.4 g/L after removal of ECMO (*P* < 0.001). Renal function parameters and cardiac ultrasound indices did not differ significantly between the period before PCI and the 24-h period after the removal of ECMO.

**Table 3:** Comparison of laboratory and cardiac function parameters before and after PCI.

**Parameter**	**Pre-PCI**	**Post-PCI**	***P*-value**
Hb (g/L)	145.5 ± 19.9	105.5 ± 21.4	<0.001
PLT (×10^9^/L)	179.8 ± 52.9	162.1 ± 52.3	0.15
eGFR	85.5 ± 24.2	86.9 ± 30.3	0.39
LVEF (%)	46.8 ± 11.6	48.1 ± 9.0	0.06
LVEDV (ml)	152.2 ± 55.8	157.1 ± 61.1	0.50
CO (L/min)	4.8 ± 1.0	4.8 ± 1.0	0.74
CI (L/min/m^2^)	2.7 ± 0.5	2.7 ± 0.5	0.76

*CI, cardiac index; CO, cardiac output; eGFR, estimated glomerular filtration; Hb, haemoglobin; LVEDV, left ventricular end-diastolic volume; LVEF, left ventricular ejection fraction; PCI, percutaneous coronary intervention; PLT, platelets*.

### Clinical Outcomes

Table 4 summarises the outcomes during hospitalisation included death (*N* = 1, 2.8%) due to bacteraemia, stroke (*N* = 1, 2.8%). No myocardial infarctions occurred during hospitalisation. Other important events included lower limb ischaemia (*N* = 2, 5.6%), lower limb deep venous thrombosis (*N* = 1, 2.8%), cannulation site haematoma (*N* = 2, 5.6%), bacteraemia (*N* = 2, 5.6%), and bleeding requiring blood transfusion (*N* = 5, 13.9%), and AKI (*N* = 2, 5.6%). One patient with AKI was treated with continuous renal replacement therapy (CRRT) due to oliguria and electrolyte disturbance, while the other patient showed no significant decrease in urine volume and was stable without CRRT.

**Table 4:** Clinical outcomes.

**Parameters**	**ECMO (*N* = 36)**
In-hospital mortality	1 (2.8%)
Re-infarction	0 (0)
Ischaemic stroke	1 (2.8%)
Limb ischaemia	2 (5.6%)
Deep venous thrombosis	1 (2.8%)
Arteriovenous fistula	0 (0)
Pseudoaneurysm	0 (0)
Cannulation site hematoma	4 (11.4%)
Acute kidney injury	2 (5.6%)
Bacteraemia	2 (5.6%)
Bleeding requiring transfusion	5 (13.9%)
Continuous renal replacement therapy	1 (2.8%)

*Data are presented as N (%)*.

*ECMO, extracorporeal membrane oxygenation*.

**Figure 1:**
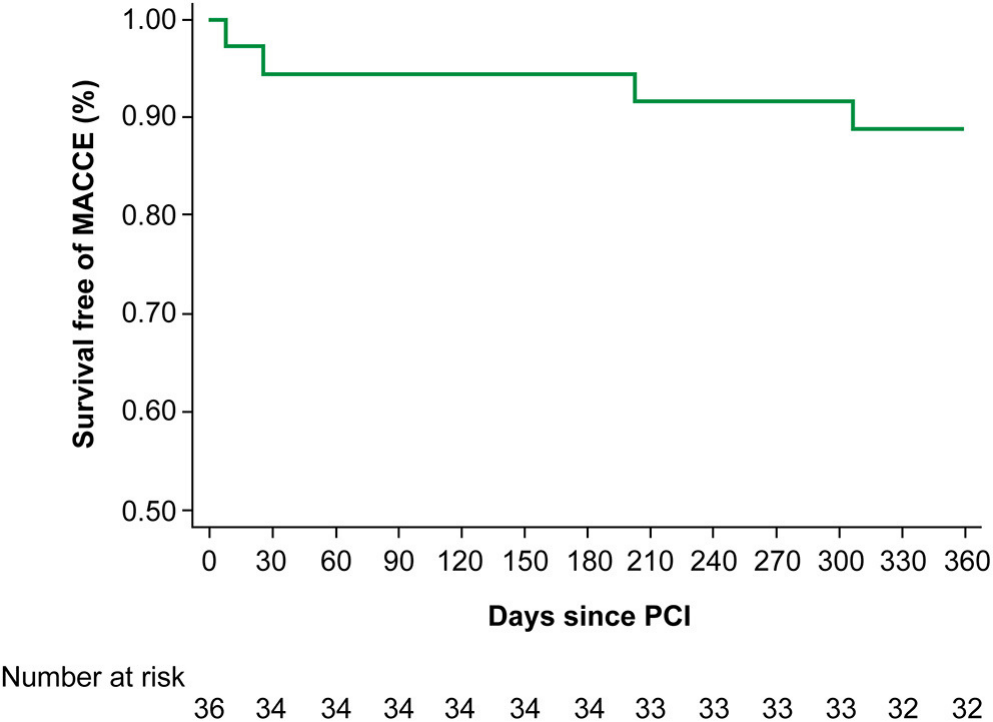
The incidence of MACCE within 12 months of PCI. MACCE, major adverse cardiovascular and cerebrovascular event; PCI, percutaneous coronary intervention.

Figure 1 illustrates the incidence of MACCEs within 12 months of PCI. One (2.8%) patient died and one (2.8%) patient had ischaemic stroke during hospitalisation, two (5.6%) patients were hospitalised for heart failure. There were no myocardial infarctions.

## Discussion

The results from 36 patients in this study suggest that using VA-ECMO during elective HR-PCI is safe and feasible, with low mortality and complication rates. For patients with complex and high-risk coronary artery disease, selective HR-PCI supported by VA-ECMO can be used as an alternative revascularisation strategy to CABG. These results are consistent with those of other single-centre studies (18–21).

Patients with complex and high-risk coronary artery disease present with the following three characteristics: first, severe coronary artery disease, such as multi-vessel disease or unprotected left trunk, chronic obstructive disease and calcification, and consequent complications; second, comorbidities including heart failure, diabetes, previous CABG, and advanced age; and third, haemodynamic changes, such as haemodynamic instability, shock, or severe left ventricular dysfunction (3, 4).

As an elective revascularisation strategy for complex and high-risk coronary artery disease, PCI revascularisation is the preferred choice for one or two coronary artery lesions with or without left anterior descending artery stenosis, whereas CABG is preferred for patients with left main coronary artery disease with a SYNTAX score ≥23, three-vessel disease without diabetes with a SYNTAX score ≥23, and three-vessel disease with diabetes (1). The results of the 10-year SYNTAX trial revealed no difference between PCI and CABG in all-cause mortality 10 years after revascularisation. In our subgroup analysis, the survival rate of patients with three-vessel coronary artery disease in the CABG group was higher than that in the PCI group. However, there was no difference between PCI and CABG in the survival rates of patients with left main coronary artery disease. Consequently, in patients with triple-vessel disease or left main artery disease, revascularisation strategy (PCI or CABG) is decided by the cardiac surgeons and interventional physicians (2). For complex high-risk coronary artery disease, overall revascularisation rates remain low irrespective of PCI or CABG. In one study, 4,414 patients with non-ST-elevation myocardial infarction (NSTEMI) were divided into low-risk, medium-risk, and high-risk groups according to the Global Registry of Acute Coronary Events score. While the rate of revascularisation in the high-risk group was significantly lower than that in the low-risk group, the overall rates of revascularisation (PCI or CABG) increased gradually with time (7). In another observational study focusing on revascularisation in patients with NSTEMI and multi-vessel coronary artery disease with diabetes complications (*N* = 29,769), only one-third of the patients underwent CABG within 6 years, and nearly half underwent PCI. While the overall revascularisation rate increased, 17.3% of patients did not receive revascularisation treatment. Additionally, while the proportion of patients who received PCI treatment increased gradually, the proportion of those who received CABG treatment remained unchanged.

It has been suggested that revascularisation (PCI or CABG) in patients with complex, high-risk coronary artery disease can improve their prognosis (5, 6). However, HR-PCI includes several challenges (3, 9): (1) There is a dearth of research data because of insufficient revascularisation rates and a lack of objective and accurate evidence supporting an optimal revascularisation strategy; (2) Interventional doctors may underestimate the benefits of revascularisation in such patients; (3) Revascularisation in coronary artery disease patients is difficult, intraoperative procedures and complications may also have serious adverse effects on haemodynamic parameters in these patients; (4) Operators are required to master techniques, such as fractional flow reserve, intravascular ultrasound, and optical coherence tomography. Therefore, a considerable number of interventional physicians may lack the required competence.

A growing body of clinical evidence suggests the usefulness of left heart assist devices as circulatory support in HR-PCI. The results of the IABP-SHOCK II trial suggested that IABP, an older circulatory assist device, was ineffective in patients with circulatory failure (22). Al-Khadra et al. evaluated non-emergency PCI with a percutaneous ventricular assist device (PVAD) and IABP support in patients with no cardiogenic shock and acute myocardial infarction, respectively. According to their findings, patients undergoing a PVAD-supported PCI exhibited significantly lower in-hospital mortality rates than patients treated with an IABP (23). ECMO can be used for powerful haemodynamic mechanical circulation support during elective HR-PCI (10, 11). When VA-ECMO is used in HR-PCI, not only the cardiac functional status of the patient, but also the severity of coronary artery disease should be fully considered by the interventional physician. Because of the risk of potentially severe hemodynamic instability in severe coronary artery disease during PCI. Therefore, in this study, in addition to patients with LVEF ≤ 35%, we considered that patients with LVEF > 35% who needed rotational atherectomy during PCI, unprotected left main disease, and two CTOs with one coronary artery severe stenosis also needed VA-ECMO support. The severity of coronary artery disease requires an indication for VA-ECMO support, and more research evidence is currently needed. Four single-centre, small-sample-size studies on VA-ECMO-supported HR-PCI reported that VA-ECMO is safe and effective as a mechanical circulation support strategy for HR-PCI. Additionally, elective HR-PCI supported by VA-ECMO is a feasible choice for patients who do not qualify for CABG—or are considered very high-risk—with good short-term and long-term prognoses (18–21). Currently, there are few clinical data on HR-PCI supported by VA-ECMO, and its benefits need to be further verified using randomised controlled trials. To implement this strategy, experienced ECMO and cardiac interventional physicians' teams are required. Compared with other percutaneous mechanical assist devices, ECMO is more difficult to operate. Additionally, its complications have affected its clinical development and patient prognosis (24). The ability to prevent and manage such complications mainly depends on the team's ability to diagnose, treat, and nurse patients on ECMO. Studies have demonstrated that an ECMO treatment of >20 critical patients per year can maintain the level of experience in ECMO treatment centres (25) and that the mortality rate in adult ECMO centres treating >30 cases per year is significantly lower than that in centres treating <6 cases per year (26).

The main complications of ECMO in this study included vascular puncture complications, lower limb ischaemia, deep venous thrombosis, bleeding, increased left ventricular afterload, acute renal injury, and infection.

This study evaluated all patients using ultrasound at the puncture site (including the femoral artery and femoral vein) before ECMO intubation. To avoid lower limb ischaemia and thrombosis, the vessel diameter should be at least 1–2 mm larger than that of the intubation cannula (27). The recommended solution for lower limb ischaemia is to place a short distal 6–8 F perfusion catheter in the ipsilateral superficial femoral or dorsalis pedis artery (24, 27). In this study, lower limb ischaemia improved after a distal perfusion catheter was placed in two (5.7%) patients. The trigger for distal reperfusion catheter placement is the 5P sign of acute limb ischaemia, characterised by persistent pain with pallor, pulselessness, paraesthesia, and paralysis. Moreover, ultrasound can be used to evaluate the blood flow in punctured blood vessels, the presence of atherosclerotic plaques, and calcification at the puncture site to avoid catheter placement in such areas. No arteriovenous fistula or pseudoaneurysm complications were observed in this study, which was related to ultrasound-guided percutaneous cannulation. Based on our experience, the placement of arterial and venous cannulae was ultrasound-guided rather than fluoroscopic. While adequate anticoagulation is required during ECMO, anticoagulation therapy carries a potential bleeding risk. Four (11.1%) patients in this study developed haematoma at the intubation site, and five (13.9%) patients received a blood transfusion due to decreased haemoglobin levels, primarily due to loss of blood in the tube, during weaning from ECMO.

In this study, IABP was used as the left ventricular decompression strategy in 44.4% of patients, while the remaining patients were supported by ECMO alone. With increases in blood flow, VA-ECMO tends to increase the left ventricular afterload. The most common devices used for left ventricular unloading during VA-ECMO are Impella (Abiomed) and IABPs, while other strategies include atrial septostomy, surgical left ventricular apical drainage, positive inotropic drugs, diuretics, or continuous renal replacement therapy (10, 11). Compared with no unloading, any unloading strategy can reduce the mortality of patients with VA-ECMO (28). In patients who undergo VA-ECMO for cardiogenic shock, there is no significant difference in haemodynamic parameters between IABP and Impella (Abiomed) in left ventricular afterload reduction. However, the use of IABP combined with ECMO may help reduce the mortality rate and improve the 180-day survival rate (29). IABP is the most commonly used left ventricular decompression device in VA-ECMO because it can be implanted percutaneously at the bedside within a short period and is easy to operate. IABP combined with VA-ECMO in patients with cardiogenic shock can significantly reduce the in-hospital and 28-day all-cause mortality rates and contribute to successful weaning from ECMO (30). For selective HR-PCI supported by VA-ECMO, the timing of IABP as a left ventricular unloading strategy requires further research.

Acute kidney injury is a prognostic complication of ECMO. Studies have reported that the rate of severe AKI requiring renal replacement therapy during ECMO is approximately 45% (31). While ECMO can improve renal function, it can simultaneously increase AKI risk. AKI in patients on ECMO support is often caused by multiple factors, such as ischaemia-reperfusion injury, inflammatory reactions, and ECMO damage to blood cells (32). A single-centre retrospective study of 2,660 patients with coronary heart disease who underwent PCI, including 1,128 patients with non-complex PCI and 1,532 patients with complex PCI, reported no difference in contrast-associated kidney injury between the two groups. This finding suggested that complex PCI does not increase the rate of contrast-induced renal injury (33). It is believed that HR-PCI does not increase contrast-associated AKI. In this study, two patients developed kidney injury post-operatively, which corresponds to a low AKI rate. This observation may be related to the short duration of ECMO and the small number of patients in this study.

The infection rate in patients on VA-ECMO registered with the Extracorporeal Life Support Organisation during 2014–2018 was 7.6% (24). The common complications of VA-ECMO infection are bacteraemia and sepsis. The infection rate increases gradually with the prolongation of ECMO support. More than 53% of patients with infection-related complications develop them within 2 weeks of ECMO initiation (26). The rate of ECMO-associated infections in patients in this study was 5.6%. The duration of ECMO support was 12.5 (3.0–26.3) h, and the relatively shorter ECMO uptime contributed to the low rate of ECMO-related infections. However, infection rate seems high given the short duration of support in this study, we reviewed the clinical data of both patients with ECMO-associated infections and found that both patients received combined IABP and one patient received combined CRRT for AKI. Therefore, the increased mechanical support may increase the risk of infection in patients.

## Limitations

This study was a single-centre retrospective study with a small sample size. Additionally, vascular complications might have been underestimated as women, whose smaller blood vessels may result in more cannulae-size-related complications, were underrepresented in this study. Based on our experience, we plan to perform a randomised controlled trial with VA-ECMO as circulating support during HR-PCI to further clarify the indications for and timing of VA-ECMO in HR-PCI (ChiCTR2100046630).

## Conclusions

Prophylactic use of VA-ECMO as a circulatory support device during elective HR-PCI is safe and feasible. Complication and MACCE rates during the use of ECMO in HR-PCI and the rate of MACCE at 1-year post-operatively were low. The optimal timing of HR-PCI using VA-ECMO requires further validation.

## Data Availability Statement

The raw data supporting the conclusions of this article will be made available by the authors, without undue reservation.

## Ethics Statement

The studies involving human participants were reviewed and approved by the Ethics Committee of the First Hospital of Lanzhou University. Written informed consent for participation was not required for this study in accordance with the national legislation and the institutional requirements.

## Author Contributions

MB, AL, CP, SH, WQ, JZ, and BZ performed the study. WQ and JZ performed the analyses. MB, AL, and CP drafted the manuscript. MB helped supervise the project. All authors contributed to the article and approved the submitted version.

## Funding

This work was supported by the Key Science and Technology Foundation of Gansu Province (No. 21YF5FA118) and the Natural Science Foundation of Gansu Province (No. 21JR7RA385).

## Conflict of Interest

The authors declare that the research was conducted in the absence of any commercial or financial relationships that could be construed as a potential conflict of interest.

## Publisher's Note

All claims expressed in this article are solely those of the authors and do not necessarily represent those of their affiliated organizations, or those of the publisher, the editors and the reviewers. Any product that may be evaluated in this article, or claim that may be made by its manufacturer, is not guaranteed or endorsed by the publisher.

## Abbreviations

AKI: acute renal injury
BARC: Bleeding Academic Research Consortium
CABG: coronary artery bypass grafting
CI: confidence interval
CRRT: continuous renal replacement therapy
CTO: chronic total occlusion
ECMO: extracorporeal membrane oxygenation
EF: ejection fraction
F: French
HR: hazard ratio
HR-PCI: high-risk percutaneous coronary intervention
EuroSCORE: European system for cardiac operative risk evaluation
IABP: intra-aortic balloon counterpulsation pump
J-CTO: CTO registry of Japan
LVEF: left ventricular ejection fraction
MACCE: major adverse cardiovascular and cerebrovascular events
MT: medical therapy
NSTEMI: Non-ST elevation myocardial infarction
PCI: percutaneous coronary intervention
PVAD: percutaneous ventricular assist device
SCr: serum creatinine
TIMI: thrombolysis in myocardial infarction
VA-ECMO: veno-arterial extracorporeal membrane oxygenation.
